# Effect of Body Mass Index on work related musculoskeletal discomfort and occupational stress of computer workers in a developed ergonomic setup

**DOI:** 10.1186/1758-2555-3-22

**Published:** 2011-10-07

**Authors:** Jasobanta Sethi, Jaspal Singh Sandhu, Vijay Imbanathan

**Affiliations:** 1 Department of Sports Medicine & Physiotherapy, Guru Nanak Dev University, Amritsar, Punjab, India; 2 Department of Sports Medicine & Physiotherapy, Guru Nanak Dev University, Amritsar, Punjab, India; 3 Department of Psychology, Laxmi Memorial College of Physiotherapy, Mangalore, India

## Abstract

**Background:**

Work urgency, accuracy and demands compel the computer professionals to spend longer hours before computers without giving importance to their health, especially body weight. Increase of body weight leads to improper Body Mass Index (BMI) may aggravate work related musculoskeletal discomfort and occupational-psychosocial stress. The objective of the study was to find out the effect of BMI on work related musculoskeletal discomforts and occupational stress of computer workers in a developed ergonomic setup.

**Methods:**

A descriptive inferential study has been taken to analyze the effect of BMI on work related musculoskeletal discomfort and occupational-psychosocial stress. A total of 100 computer workers, aged 25-35 years randomly selected on convenience from software and BPO companies in Bangalore city, India for the participation in this study. BMI was calculated by taking the ratio of the subject's height (in meter) and weight (in kilogram). Work related musculoskeletal discomfort and occupational stress of the subjects was assessed by Cornell University's musculoskeletal discomfort questionnaire (CMDQ) and occupational stress index (OSI) respectively as well as a relationship was checked with their BMI.

**Results:**

A significant association (p < 0.001) was seen among high BMI subjects with their increase scores of musculoskeletal discomfort and occupational stress.

**Conclusion:**

From this study, it has been concluded that, there is a significant effect of BMI in increasing of work related musculoskeletal discomfort and occupational-psychosocial stress among computer workers in a developed ergonomic setup.

## Background

Work related Musculoskeletal Disorders (WMSD) are the class of musculoskeletal disorders that include damage of tendons, tendon sheaths, and synovial lubrication of tendon sheaths, and related to bones, muscles, nerves of hands, wrists, elbows, shoulders, neck and back. Other commonly used terms include Ergonomic Disorders, Cumulative Trauma Disorders (CTD) and Repetitive Strain Injuries. These disorders develop gradually over a period of week, months or even years due to repeated exertions and movements of the body. These musculoskeletal disorders belong to a collection of health problems that are more prevalent among the working class than general population [1]. Work related musculoskeletal disorders constitute a major source of employee disability and lost wages. Thus, active surveillance of WMSD should continue an essential component in an ergonomic program used to control WMSD and reduce human suffering, lost workdays and wages, and compensation claims.

The changes brought about by the development of Video Display Terminal (VDT) technology may have contributed to the increase in CTD associated with VDT use. Office workers in United States have experienced an increase in CTD since 1986 [2]. Additional factors, such as increased awareness on the part of office workers and physicians as well as better recording of CTDs may also have contributed to this increased incidence [3]. Musculoskeletal symptoms and impairment affect approximately 29.7 to 32.6% of the population of the United States, and low back pain is the most frequent disorder to be involved. The incidence of neck disorders as a source of musculoskeletal impairment or disability is second to lower back disorders [4].

Pressure on soft tissues caused by external surfaces termed as contact stress or strain. Psychosocial stress is defined as organizational or interpersonal factors resulting in increased actual or perceived stress [5]. Stress in office work of VDT operators has two components: the first is associated with introducing new technology inherent in the use of VDT; the second is associated with the job demands and job position. The stress contributed by new technology is often transient. Electronic monitoring, however, is a technology related stress that may not be transient. Electronic monitoring has been used in jobs as diverse as truck drivers, nurses and telephone operators [6]. Occupational-psychosocial Stress (OS) in VDT operators may be related more to the total job and organizational structure than the VDT themselves. Some research has reported that job level is a better indicator of stress than VDT use [7] for example, those with better jobs are more likely to be able to set their own priorities. OS has been linked to jobs that include rigid work procedures, lack of social support, monotony and insecurity. Many individuals in their jobs express dissatisfaction with their position.

Many factors have already been identified that cause WMSD and OS. Ergonomic workstation helps in the reduction of WMSD and stress as well as throws an opportunity to have better work performance for better and faster industrial production. However, another factor is the overweight or obesity, which influences the WMSD and OS even in a developed ergonomic setup. The current study will help in providing information of awareness of overweight and effect of overweight on sustained work in an upright position as well as to quantify different dimensional work related musculoskeletal discomfort and occupational stress of computer professionals with correlation to their body mass indices in a developed ergonomic setup.

## Materials & method

### Inclusions

Subjects with the age group between 21 to 35 years, working in a developed ergonomic setup (i.e. Computer workstation: ergonomic design and anthropometric data of workers [8-12]; Monitor size: 17 inches, position of monitor: arms length distance (20-26 inches) with 10-20 degree tilt (as per individual preference), top of the viewing screen is at eye level when the user is sitting in an upright position (Bifocal wearers may need to lower the monitor to a couple of inches), viewing angle 40 degree with reduced glare, keyboard position: flat or neutral, mouse kept at side of key board, document holder (if required): preferably, at side of the monitor, Chair with 5 point base with casters, 15-22 inches adjustable seat height, (for individual convenience) feet rest flat on floor (footrest used if necessary), Seat size: 16.9 inches depth, 17.7 inches width, angle 0-4 degree with a waterfall front edge. Backrest size: 17.7 inches high, 14.2 inches width, adjustable lumbar support; 5.9 to 9.8 inches, backrest tilt/recline: adjustable 15 degree forward and backward (as per user preference), angle between backrest and seat pan: 90 degree or greater, arm rest: 10 inches high, 9.5 inches length, 2 inches width, removable/ height-adjustable arm rest (as per individual preference), well padded armrests, not used for slouch, Table: height of the table: 30 inches (for better leg room below the keyboard and mouse tray), height of key board and mouse tray: 26.5 inches below elbow height, Knee room: height (26 inches), width (20 inches), depth (15 inches). Anthropometric data of workers: head in straight/erect position, shoulders: relaxed (bilateral), shoulder abduction angle is less than 20 degree for working with mouse, shoulder-elbow angle: 90 degree, wrist in neutral position (fore arm and hand in a straight line, hip-torso angle: 90 degree, thigh-leg angle: 90 degree, leg-foot angle: 90 degree) as well as checked with OSHA Ergonomic Solutions: Computer Workstations eTool - Evaluation Checklist [13], those present during data collection, educational qualification - professional degree and above in engineering and computer science (upper-I socioeconomic status) [14-16], work experience of more than one year and willingness towards participation have been included for study.

### Exclusions

Part-time workers, subjects suffering from chronic illness and those underwent major surgery, eye problems, post-traumatic stiff joints, fixed deformity, weakness and paralysis were excluded.

### Procedure

Subjects were selected by simple random sampling based on inclusion and exclusion criteria along with fulfillment of OSHA Ergonomic Solutions: Computer Workstations eTool - Evaluation Checklist [13] with a written consent signed by them for participation in this study. All the respondents completed the questionnaires anonymously, recording their individual ID number. No expenditure was inflicted on the cases, and all the personal records were considered confidential. The study was started after receiving approval from the institutional ethical committee. Body Mass Index (BMI) [17] was calculated by taking the ratio of the subject's height (in meter) and weight (in kilogram) i.e. (weight/ (height)^2^. Work related musculoskeletal discomfort was assessed by Cornell University's Musculoskeletal Discomfort Questionnaire (CMDQ) [18,19] and occupational-psychosocial stress (role overloads, role ambiguity, etc.) was assessed by Occupational Stress Index (OSI) [20,21] and the score was taken for calculation. The association was checked between different body mass indices and the scores of musculoskeletal discomfort and the occupational stress index.

## Data Analysis

The data was analyzed for statistical significance by using the statistical package of social science (SPSS 11.0 Systat 8.0) software. The effect of BMI on WMSD and OS was analyzed by ANOVA. Separate Chi square analysis was done to associate BMI with OSI scores. Also a multivariate discriminant functional analysis was done to predict the BMI based on the study parameters (WMSD & OS) and OSI components.

## Results

**Figure 1:**
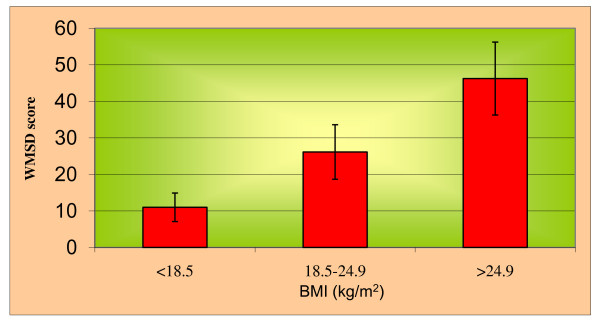
**Association of Body Mass Index (BMI) with Work Related Musculoskeletal Discomfort (CMDQ Score)**.

**Figure 2:**
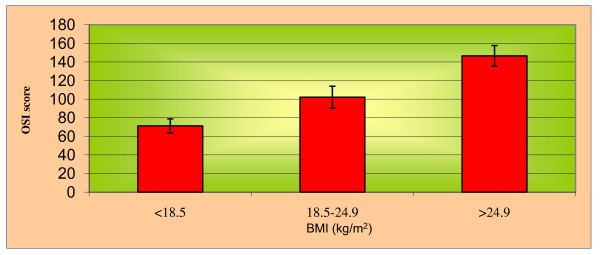
**Association of Body Mass Index (BMI) with Occupational-psychosocial Stress (using OSI score)**.

Maximum 60% of subjects were noted in the age group of 31-35 years with involvement of WMSD and OS (Table 1), whereas 64% of subjects were noted in high BMI group (Table 2). Maximum CMDQ score was noted in the overweight group (Mean, 46.23) followed by normal weight group (Mean, 26.13) and underweight group (Mean, 11.00), because overweight may contribute to increasing work related musculoskeletal disorders due to more weight loads on tissues. Significant association of BMI with CMDQ score (F = 136.137, P < 0.001; Table 3 & Figure 1) and OSI score (F = 422.295, P < 0.001; Tables 4 and 5 & Figure 2) has been found in this study. This shows that, high BMI group perceives a high level of WMSD and OS. Multivariate Discriminant Function Analysis was done to predict the BMI based on two parameters (Table 6). It has been noted that as the BMI increases, the CMDQ score significantly increases (P < 0.001), and OSI score also increases (P < 0.001). A Multivariant discriminant function analysis was done to predict the BMI over OSI sub components in which significance (P < 0.001; Table 7) has been seen with the role overload, unreasonable group pressure, responsibility and strenuous working conditions only.

**Table 1:** Subject distribution with their age group

Age in years	Subjects	
	
	Number	Percentage
21-25	14	14.0

26-30	26	26.0

31-35	60	60.0

Total	100	100.0

**Table 2:** Body Mass Index (BMI) distribution (kg/m^2^)

BMI (kg/m^2^)	Number	Percentage
< 18.5 (under weight)	20	20.0

18.5-24.9 (normal weight)	16	16.0

> 24.9 (over weight)	64	64.0

Total	100.0	100.0

Mean ± SD	24.58 ± 4.33	

**Table 3:** Association of Body Mass Index (BMI) with Work related Musculoskeletal Discomfort (CMDQ Score)

BMI (kg/m^2^)	CMDQ score	
	
	Range	Mean ± SD
< 18.5	5-18	11.00 ± 3.91

18.5-24.9	13-40	26.13 ± 7.46

> 24.9	26-82	46.23 ± 9.98

Total	5-82	35.97 ± 16.87

Inference	As the BMI increases the CMDQ score increases significantly with F = 136.137*	

* Significant (p < 0.001)

**Table 4:** Association of Body Mass Index with Occupational-psychosocial Stress (OSI score)

BMI (kg/m^2^)	OSI score	
	
	Range	Mean ± SD
< 18.5	61-97	71.25 ± 7.50

18.5-24.9	92-144	102.06 ± 11.81

> 24.9	107-166	146.52 ± 11.15

Total	61-166	124.35 ± 32.84

Inference	As the BMI increases the OSI score increases significantly with F = 422.295*	

* Significant (p < 0.001)

**Table 5:** Association of Occupational-psychosocial Stress with Body Mass Index

OSI	BMI (kg/m^2^)			Total
		
	< 18.5	18.5-24.9	> 24.9	
≤ 76 Mild stress	17 (85.0%)	-	-	17%

77-152 Moderate stress	3 (15.0%)	16 (100.0%)	47(73.4%)	66%

153-230 Severe stress	-	-	17 (26.6%)	17%

Total	20	16	64	100

Inference	Higher OSI score is significantly associated with higher BMI ( > 24.9 kg/m^2^) with χ^2 ^= 36.412*			

* Significant (p < 0.001)

**Table 6:** Multivariate discriminant function analysis to predict the BMI based on two study parameters

Study parameters	Percentage of correct classification of BMI	Wilk's Lambda	χ^2 ^value
1. CMDQ score	86.0	0.263	129.67*

2. OSI score	97.0	0.103	220.467*

3.ALL	99.0	0.022	365.024*

* Significant (p < 0.001)

**Table 7:** Multivariate Discriminant function analysis to predict the BMI based on OSI components

OSI components	Percentage of correct classification of BMI	Wilk's Lambda	χ^2 ^value
1. Role over load	96.0	0.120	205.765*

2. Role ambiguity	96.0	0.289	120.560

3. Role conflict	93.0	0.157	179.864

4. Unreasonable group and Pol. Pressure	92.0	0.107	217.158*

5. Responsibility for persons	87.0	0.094	229.334*

6. Under participation	63.0	0.803	21.336

7. Powerlessness	66.0	0.681	37.209

8. Poor peer relation	73.0	0.675	38.105

9. Intrinsic impoverishment	76.0	0.457	75.950

10. Low status	53.0	0.788	23.102

11. Strenuous working condition	91.0	0.097	225.950*

12. Un-profitability	90.0	0.193	159.541

All	97.0	0.011	412.580*

* Significant (p < 0.001)

## Discussion

Computer worker's health is foremost important for better productivity of any IT or BPO Company. Correct ergonomic setup, frequent rest, stretching and strengthening exercises may reduce few degrees of physiological and psychological load in the body, but at the same time importance has to be given for reduction of body weight in their sedentary working life otherwise it might lead to serious work related musculoskeletal disorders and occupational-psychosocial stress in due course of time.

An effort has been made here to find out the influence of BMI over CMDQ and OSI scores of the subjects in a developed ergonomic setup (Computer workstation: ergonomic design and anthropometric data of workers) [8-12]. In this study, 100 computer workers of different BMI were randomly selected (those who have given consent for participation) with fulfillment of OSHA Ergonomic Solutions: Computer Workstations eTool - Evaluation Checklist [13].

Evaluation of WMSD has already been studied by many authors in different Indian cities on computer professionals [22-25]. The CMDQ [18,19] is a reliable and valid tool, which has been taken here for investigation as well as already been used in foreign [26,27] and Indian [24] studies for measurement of WMSD of computer professionals. The WMSD also has been studied in various other occupations in Indian population [28-39].

In this study, maximum percentage of subjects was noted in the age group of 31-35 years as well as under high BMI. Computer workers with high BMI were found to be at risk with more WMSD and occupational-psychosocial stress, because over weight could be the factor to contribute in increasing of physiological and mechanical load on tissues. Relative disk pressure is being experienced during sitting with various inclinations of the back support. Intra-diskal pressure of the nucleus pulposus acts as a load transducer and indicates the magnitude of axial loading on the spinal column and the increased pressure indicates a greater muscular effort in maintaining the posture and hence a larger stress on spinal column [40].

Overweight yields a decreased postural stability and potentially negative impact on control of upper limb movements but its effect on control of balance imposes constraints on goal-directed movements. From a clinical perspective, obese individuals might be less efficient and more at risk of injuries than normal individuals in a large number of work tasks and daily activities especially requiring upper limb movements performed from an upright position [41].

Here increased CMDQ score was significantly associated with high BMI (P < 0.001, F = 136.137). Hence, high BMI has a definite influence in increasing WMSD even in a developed ergonomic setup. The finding inferred that high BMI computer professionals were prone to musculoskeletal disorder at work. This could be because of the body tissues are with stress load due to increased BMI which contributes to musculoskeletal discomforts. In support of this finding Shiri *et al. *[42] confirmed about the association between weight-related factors and the prevalence of Low Back Pain. Sjolie [43] too reported a significant correlation between high BMI and low back pain due to lesser flexibility, especially poor hip mobility. Furthermore, longer the time period they spent before the computer, higher the tissue load they receive on different body parts, which may further aggravate in case of high BMI. In addition, IJmaker *et al. *[44] confirmed an incidence of WMSD in different body parts of office workers due to their long time exposure to the computers. The time factor was dependent on speed and accuracy of work, which could be slow in case of high BMI computer professionals forcing them to experience more WMSD.

Occupation related psychosocial stress among working population is drastically increasing worldwide. Stress at work has become an integral part of everyday life. OSI with its 12 sub-components has been used in this study for evaluation of occupational-psychosocial stress (or occupational stress) among computer workers. OSI developed by Srivastava and Singh [20,21] has been commonly used for research in Indian context [45-51].

Overweight has got an impact on occupational-psychosocial stress. Because of repetitive movements of upper limbs, completion of a certain task in a stipulated time period, competition with fellow colleagues put the overweight and obese workers in a major occupational stress. The overweight impact may contribute in functional strength of the body in a continuous sedentary task where the ability of performance compromised to some extent. In a study Riddiford *et al. *[52] has reported that obese children spent significantly more time periods during all transfer phases of the chair raising task compare to non-obese children and thereby lower limb functionality in young obese children was impeded, when they move their greater body mass against gravity. Here it has been found that overweight workers face moderate to severe occupational stress as compared to their moderate built colleagues in a stressful work environment.

Stress in office work of VDT operators are due to introduction of new technology, inherent in the use of VDT, job demands and job position. Stress in VDT operators may be related more to the total job and organizational structure than to VDT themselves. Job's level is a better indicator of stress than VDT use, for example, those with better jobs are more likely to be able to set their own priorities. Stress has been linked to jobs that include rigid work procedures, lack of social support, monotony and insecurity. In this study psychosocial factors have been checked among computer workers from a single socioeconomic status (i.e. upper I) [14-16] with high professional qualification, high earning and well to do family background. It helped in unbiased assessment of occupational-psychosocial stress claiming the impact of different BMI, since there were no other levels of socioeconomic status included.

Role over load, unreasonable group pressure, responsibility for persons, strenuous working condition has been found significant (P < 0.001) association with high BMI. This could be due to the competitive task required day by day in growing industries where the overweight computer professionals face such difficulties in stressful computing job. Previous research has focused on overall association between occupational stress and BMI. Sedentary office workers in a stressful job with high BMI will have more eating behavior, thereby they are more prone to have a weight gain which leads to obesity as reported by Kivimaki *et al. *[53] adding further occupational stress. In contrast, the weak association also has been seen between BMI and Occupational Stress of aggravated scores as reported by Kouvonen *et al. *[54].

Here, the increased OSI score has been significantly associated with high BMI (P < 0.001, F = 422.295) computer workers in a developed ergonomic setup. Hence, high BMI has a definite influence in increasing occupational-psychosocial stress. This study is confirmed by Ostry *et al. *[55] exploring the significant association exists between BMI and Occupational-Psychosocial Stress.

## Conclusion

It can be concluded by stating that, there is a significant effect of BMI in increasing of WMSD and occupational-psychosocial stress. This study provides the insight to the Clinicians and Ergonomists about the relationship between BMI and WMSD, occupational stress in order to formulate well designed training program to avoid overweight for making the computer professionals fit at their sedentary work and free from occupational injury and stress.

## Suggestion

Further study is required to find out the effect of BMI on followings:

1) Equal number of male and female population can be taken for the study.

2) Visual problems can be taken into consideration separately

3) Mental stress can be added along with occupational stress.

## Abbreviations

BMI: Body Mass Index; WMSD: Work-related Musculoskeletal Discomforts; OS: Occupational-psychosocial Stress; OSI: Occupational Stress Index; VDT: Video Display Terminal.

## Competing interests

The authors declare that they have no competing interests.

## Authors' contributions

Authors JS and VI have made substantial contributions to conception and design, acquisition of data, analysis and interpretation of data, JS and JSS have been involved in drafting the manuscript, revising it critically for important intellectual content, and all the authors read and approved the final manuscript.
